# Impact of age-related neuroglial cell responses on hippocampal deterioration

**DOI:** 10.3389/fnagi.2015.00057

**Published:** 2015-04-29

**Authors:** Joseph O. Ojo, Payam Rezaie, Paul L. Gabbott, Michael G. Stewart

**Affiliations:** ^1^Department of Life Sciences, The Open UniversityWalton Hall, UK; ^2^Department of Neuropathology, Roskamp InstituteSarasota, FL, USA

**Keywords:** aging, microglia, astrocytes, neuroinflammation, hippocampus, cognition

## Abstract

Aging is one of the greatest risk factors for the development of sporadic age-related neurodegenerative diseases and neuroinflammation is a common feature of this disease phenotype. In the immunoprivileged brain, neuroglial cells, which mediate neuroinflammatory responses, are influenced by the physiological factors in the microenvironment of the central nervous system (CNS). These physiological factors include but are not limited to cell-to-cell communication involving cell adhesion molecules, neuronal electrical activity and neurotransmitter and neuromodulator action. However, despite this dynamic control of neuroglial activity, in the healthy aged brain there is an alteration in the underlying neuroinflammatory response notably seen in the hippocampus, typified by astrocyte/microglia activation and increased pro-inflammatory cytokine production and signaling. These changes may occur without any overt concurrent pathology, however, they typically correlate with deteriorations in hippocamapal or cognitive function. In this review we examine two important phenomenons, firstly the relationship between age-related brain deterioration (focusing on hippocampal function) and underlying neuroglial response(s), and secondly how the latter affects molecular and cellular processes within the hippocampus that makes it vulnerable to age-related cognitive decline.

## Introduction

### Aging

Aging is a natural biological process that is universal and intrinsic to all animals. It is progressive, time-dependent, and deleterious in nature and results ultimately in physiological decline, but exhibits variability between individuals (Lister and Barnes, 2009). With respect to the brain, in humans and rodents, one of the most common occurrences indicative of the normal aging process, is an association with cognitive decline, even in many cases without a major pathological component. Due to advances in healthcare, there has been an increase in the life expectancy of humans, with the fastest increasing age group being amongst those who are 85 years and over. According to the office of national statistics (USA), by 2034 it is projected that 5 per cent of the total population will be over 85 years. Since aging is a major risk factor, albeit not a cause, there is a greater propensity to develop dementia as we age (Bishop et al., 2010). Thus understanding the major underlying mechanisms that accompany the normal process of aging will be important to dissect the processes that amplify the vulnerability of the brain to the development of age-related cognitive decline.

### Normal Aging and Pathological Aging

Defining the boundaries of normal physiological change as distinct from pathological conditions poses a major challenge in the study of the brain aging process. For example, in Alzheimer’s disease, an age-related neurodegenerative condition characterized by progressive decline in cognitive function, there are widespread depositions of amyloid plaques and neurofibrillary tau pathology accompanied by aberrant neuroinflammatory responses in the brain. These changes have also been consistently reported in some population of healthy non-agerians or centenerians (Braak and Braak, 1991). The dramatic increase in the probability of aged individuals (>85 years) suffering from late onset-sporadic neurodegenerative diseases like AD is an evidence of the impact of aging. (Mattson and Magnus, 2006). This raises the question as to whether aging might represent (i) a very early indicator of a pathological process, whereby aging is prodromal to age-related neurodegenerative diseases and occurs along a continuum, albeit at different rates in individuals. Or (ii) whether clear distinct differences in some histopathological findings between normal and pathological aging are purely associated with an independent activated pathway involving genetic predisposition, separate molecular and cellular mechanisms, as well as susceptibility to environmental factors (Dickson et al., 1992; Mattson and Magnus, 2006). In this review we will explore the role of one common mechanism seen in normal and pathological aging (i.e. neuroglial/neuroinflammatory responses) and its impact on specific aspects of brain function.

## Evidence of Deficit in Hippocampal Function in Aging

The normal aging process is associated with conserved pathways that are affected early, before more widespread decline (Braak and Braak, 1991). One such region, that is very vulnerable and susceptible to hallmark features of age-related neurobiological alterations is the hippocampus. The hippocampus is embedded deep within the medial temporal lobe of the brain and is particularly important because of its key role in the formation of episodic-declarative memory and cognitive function.

A number of studies in humans have correlated memory impairment in apparently healthy elderly individuals with features of medial temporal lobe amnesia (Craik and Jennings, 1992; Golomb et al., 1993; Petersen et al., 2009). For example, Golomb et al. (1993) found that elderly individuals with hippocampal atrophy performed poorly on tests for declarative memory. Also neuroimaging and cognitive neurophysiological studies that measure cerebral blood flow during encoding and recognition of faces have reported deficits in memory performance in healthy elderly subjects associated with hypoactivity in the hippocampus (Grady et al., 1995). This age-related memory deficits can be primarily attributed to a functional impairment in medial temporal lobe encoding of new information, which serves as a basis for recall/recognition, rather than a deficient medial temporal lobe function during the retrieval of information (Gallagher and Rapp, 1997). In support of this hypothesis, Schacter et al. (1996) reported that during a word-stem completion task by elderly subjects, hippocampal activation in both young and aged groups were similar during successful recall of studied words. However, it is noteworthy to mention that this age-related impairment is not evident in all elderly individuals, probably due to different levels of resilience and cognitive reserve. Another common finding in age-related cognitive neuropsychological studies was that during memory retrieval or recognition, older individuals also showed bilateral prefrontal cortex activity while younger matched pairs showed right prefrontal cortical activity (Aine et al., 2011). These cortical regions have been associated with a role in executive functioning and other higher cognitive functions. The changes in cortical activity have been attributed to a process of compensation or recruitment of other brain regions to help maintain good performance following age-related hypoactivity in the hippocampus, and may also signal a surrender of other cortex-dependent higher cognitive functions, such as executive function of attention, abstract thinking, and daily task planning. In aged non-human primates there is also considerable body of evidence to associate hippocampal deterioration with cognitive decline. For example, the activation of the hippocampus is reduced when aged adults perform memory related tasks dependent on the medial temporal lobe (Small et al., 2004). Reduced blood flow in the hippocampus was demonstrated also in aged rhesus monkeys, accompanied by a decrease in expression of activity regulated cytoskeletal (Arc) protein, an indicator of neuronal activity (Small et al., 2004). The development of behavioral tests geared towards the evaluation of cognitive changes across the lifespan in rodents has aided testing for impaired cognition in aging. One widely used rodent model dependent on the integrity of the hippocampus, the Morris Water Maze (Morris et al., 1986) capitalizes on the observation that experimental tasks measuring visuospatial learning (a reference associated property of declarative memory) are also sensitive to aging in rodents (Rapp et al., 1987). In this hippocampal-dependent task, rats are trained to find a hidden platform below the surface of the tank based on external cues. Numerous studies have reported that young rats can learn to swim directly to the escape platform after relatively few training trial runs (Rapp et al., 1987; Zyzak et al., 1995; Rapp and Gallagher, 1996). Aged rats (>20 months of age) on the other hand exhibit neurobehavioral deficits in this pre-learned task that are qualitatively similar to the effects of hippocampal damage, and occur independent of decline in sensorimotor/motivational functions (Rapp et al., 1987; Zyzak et al., 1995; Rapp and Gallagher, 1996).

## Glial Cell Responses in the Brain

Inflammation in the brain is defined by upregulated astrocyte and microglial cell reactivity coupled with increased levels of circulating cytokines such as TNFα, IL-1β, IFN-g (Lynch, 1998a,b; Lynch and Lynch, 2002; Streit et al., 2004). Although the brain has long been described as an immunologically privileged organ, in comparison to the peripheral system its immunological properties *per se* are not as robust. For example it lacks a lymphatic system to capture a potential antigen threat, its expression of major histocompatibility complexes (MHC I and II) is exceptionally low and the specialization of the blood brain barrier (BBB) also makes it difficult for infiltrating cells to permeate the brain parenchyma under normal conditions (Lynch, 2010). Nonetheless it is clear from studies of aging and age-related neurodegenerative disease that one of the major common hallmarks is an underlying neuroinflammatory response. Knowledge of these responses are therefore needed in order to further understand the cause and subsequent effect of these potentially damaging inflammatory changes that could be a pivotal driving force in the process of brain aging and related diseases. In this review we discuss the current major topics as they relate to age-related neuroinflammation in the “hippocampus” with a major focus on microglia responses.

### Astrocytes and Microglia

In the brain protoplasmic, fibrous and radial glia astrocytes located in the gray and white matter and axis of the ventricles respectively, by far outnumber neurons and are amongst the most numerous populations of glial cells in the brain. They perform a wide range of adaptive functions in normal brain physiology, such as: maintenance of BBB, regulation of ion homeostasis, synthesis and secretion of trophic/inflammatory factors, cell/tissue repair and regeneration, neurotransmitter uptake, lipid synthesis, synaptic transmission and regulation of synaptic density. In response to acute injury, astrocytes undergo cellular alterations including swelling, hypertrophy (astrogliosis) and proliferation (astrocytosis), characterized by increased expression of cytoskeletal protein GFAP, metallic impregnation and ultrastructural examination.

Microglia cells account for 10% of total glial cell population in the brain. They are referred to as resident macrophages and representative of the brains innate immune system. Their expression of MHC antigens, T- and B-Lymphocyte markers and other immune cell antigens, in the relatively immune privileged central nervous system (CNS) couples microglia to the adaptive immunity mediated by lymphocytes. Microglia are the first barrier of defense in the CNS, and have a ubiquitous distribution in the brain parenchyma, continuously surveying their microenvironment through their highly motile processes (Rezaie, 2007). Microglia predominate in gray matter, with high concentrations in the hippocampus and substantia nigra (McGeer et al., 1988; Lawson et al., 1990), and with a somewhat heterogeneous population in different regions of the brain (Carson et al., 2007).

There are two major distinct populations of microglia cells in the brain. (i) Short-lived, frequently replaced microglial cells derived from circulating monocytes /macrophage sources, that are concentrated in perivascular and some parenchyma regions (Kennedy and Abkowitz, 1997; Vallières and Sawchenko, 2003), and (ii) long-lived resident microglia cells which are abundant in all CNS parenchyma (Kennedy and Abkowitz, 1997; Vallières and Sawchenko, 2003).

Quiescent microglia cells, when not challenged are characterized by a small cell body, ramified process/morphology with weak expression of associated cell surface marker antigens. Upon activation in response to stimuli, activated microglia are considered to be initially neuroprotective/reparative in nature in their activity, playing vital roles in supporting and maintaining neuronal function, homeostasis and survival in normal and pathological microenvironment (von Bernhardi et al., 2010). Upon activation they undergo an initial dramatic morphological change that includes enlargement of the cell body and shortening of cellular processes. This is swiftly followed by proliferation and migration to the lesion site along a chemokine gradient. Proliferating microglia cells shield injury sites, phagocytose potentially deleterious tissue debris or dying cells, release cytokines and secrete neurotrophic factors to promote tissue repair and support growth of damaged neurons.

However, when microglia are dysregulated, their overactivation can be detrimental and result in a continuum of changes (Block and Hong, 2005; Town et al., 2005). Uncontrolled microglia responses may be harmful to survival of injured neurons if their activation supersedes threshold of tolerability, resulting in damage rather than a defensive sentinel role afflicted by excessive neuroinflammation (O’Keefe et al., 2002; Rezaie, 2007; Varin and Gordon, 2009). This sequelae of events briefly involves uncontrolled phagocytosis, induction of T-cell response, secretion of pro-inflammatory neurotoxic molecules and short-lived potentially cytotoxic species, such as nitric oxide (NO) and reactive oxygen species (ROS) which inevitably contribute to oxidative stress and mitochondrial dysfunction.

These microglia phenotypes have been speculated to be somewhat similar or analogous to the innate immune response of macrophages in the periphery, characterized by their adaptation of different activation states, involving upregulation of specific markers, in response to different signals (Gordon, 2003; Mosser, 2003; Saijo and Glass, 2011; Varnum and Ikezu, 2012). This classification is broadly described as the M1 pro-inflammatory phenotype or classical activation state, induced by Th1 cell derived cytokine IFN-γ, and identified by upregulation of TNFα, IL-1β, iNOS, and the distinct M2 anti-inflammatory/neuroprotective pathway or alternate activation state. The latter induced by the Th2 cell derived cytokines, IL4, IL5, IL10, IL13 and identified by the upregulation of mRNA expression of arginase I, mannose receptor, chitinase 3-like 3 (YM1) and found in inflammatory zone-1. M2 phenotype is characterized by the inability to produce NO and other cytotoxic/proinflammtory compounds, inefficiency at antigen presentation and stimulation of T cell proliferation.

### Astrocyte and Microglia Responses in the Aged Brain: Changes in Morphology, Density, Phenotype, Proteomic and Gene Profiles

**Figure 1:**
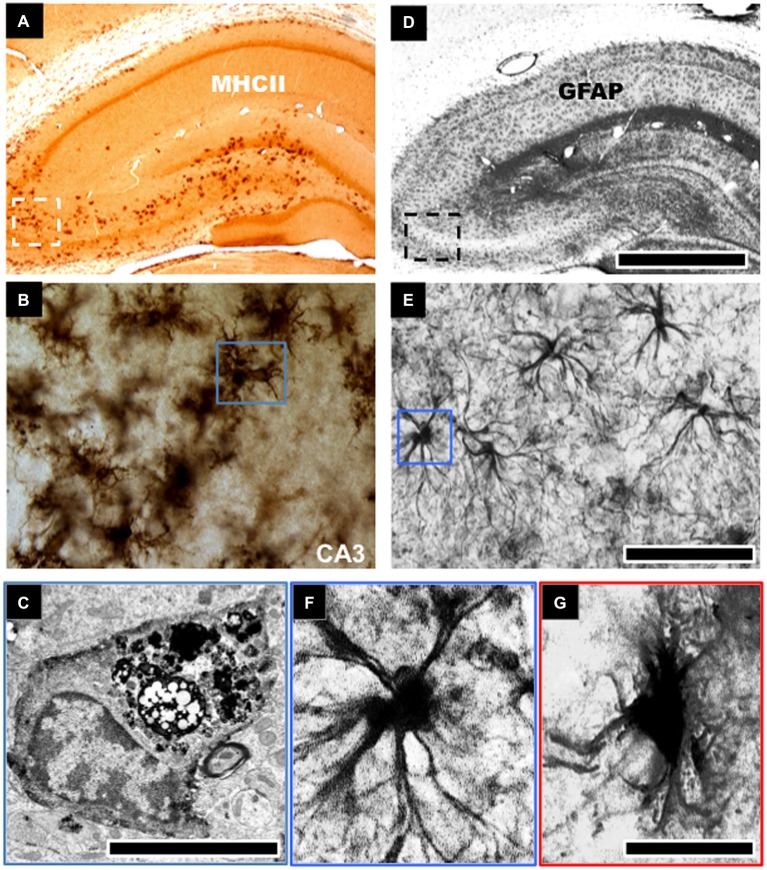
**Morphological changes to astroglial and microglial cells in the aged hippocampus**. Robust activation of microglial cells typified by an increase in cell density and expression of cell surface markers, such as, major histocompatibility complex class II (MHCII) is evident in the aged hippocampus of rodents **(A,B)**. This is notable in the CA3 and dentate gyrus regions of the hippocampus involved in learning and memory **(A)**. These microglial cells show intense electron dense staining for phagolysosomes following electron microscopic examination **(C)**. Astroglial cells in aged hippocampus become hypertrophic and increase their expression of glial fibrillary acid protein (GFAP), a marker of astrogliosis **(D,E)**. These cells demonstrate an enlarged cell body and reduced cellular processes **(F,G)**. Scale bar in **(D)** represents 733 μm in **(A,D)**; 65 μm in **(B,E)**; and 7.25 μm in **(F,G)**. Scale bar in **(C)** represents 1.25 μm.

GFAP protein and mRNA levels of resident populations of astrocytes are known to become elevated in aged rodent brains, notably in the hippocampus (Goss et al., 1991; Nichols et al., 1993; Kohama et al., 1995; Ojo et al., 2011). Histological studies have noted age-related changes in morphological characteristics of astrocytes in the hippocampus (see Figures 1D–G). These cells were described as being “fibrous” in nature, albeit without appreciable numerical changes with age (Geinisman et al., 1978; Hughes and Lantos, 1987; Mandybur et al., 1989; Bronson et al., 1993; David et al., 1997; Long et al., 1998). Thus it appears that with age the primary astrocytic change appears to be hypertrophy, rather than hyperplasia i.e., increase in cell numbers, and this is consistent in both humans and rodents. However, it is also worth mentioning that some few studies have also reported decreases in astroglial density with age (Nishimura et al., 1995; Cotrina and Nedergaard, 2002; Wu et al., 2005). Such discrepancies could be attributed to the use of GFAP as a universal marker of astrocytes. GFAP does not adequately delineate the very complex structure of individual astrocytes, which has extremely elaborate cellular processes (Rodríguez et al., 2014). This is further complicated by the heterogeneity in subpopulations of GFAP positive astrocytes between different regions of the brain (Kimelberg, 2004). Microarray analysis of the astrocyte transcriptome in the aging brain following isolation by laser capture microdissection has also shown dysregulation of genes associated with actin cytoskeleton, proliferation, apoptosis, and ubiquitin-mediated proteolysis, including altered regulation of intracellular signaling pathways, such as, insulin growth factor signaling, phosphatidylinositol 3-kinase (PI3K)/Akt, and mitogen-activated protein kinase (MAPK) pathways (Simpson et al., 2011).

In contrast with the astrocyte response, significantly more studies have been conducted to investigate the role of microglia cell response to brain aging. Increased expression of MHCII activated/primed microglia marker, and upregulated phagocytic activity has been shown by electron dense inclusions in microglia of advanced aged animals (Peters et al., 1991; Ogura et al., 1994; Sheffield and Berman, 1998; Ojo et al., 2011; see Figures 1A–C). Several *ex vivo* studies of the aged rat and human hippocampus have demonstrated morphological changes in microglial cells from a ramified to an activated state with thicker processes and larger cell somata. This is accompanied by an increase in the expression pattern of pro/inflammatory and antigen presenting markers like CD11b, MHC-II, IL-1β (and its receptor IL-1RI), IL-6, IL-18, TNFα and a decrease in the levels of anti-inflammatory cytokine markers IL-4, IL-10 and TGFβ (Lawson et al., 1990; Htain et al., 1994; Ogura et al., 1994; Streit, 2002; Godbout et al., 2005; Maher et al., 2005; Nolan et al., 2005; Griffin et al., 2006; Lyons et al., 2009). Jimenez et al. classified this age-dependent response as a switch from the alternative M2 to the classical M1 phenotype (Jimenez et al., 2008). Moreover, with age, activated/primed MHCII+ microglia cell density increases dramatically within the hippocampus in rodents (Perry et al., 1993; Ogura et al., 1994; Ojo et al., 2011), and microglia cells derived from 24-month old rodent brains also demonstrate greater proliferative capacity than cells derived from 12 month-old or younger animals (Rozovsky et al., 1998).

Reports *in vitro* have also demonstrated that microglia cells obtained by cell sorting from 18 month-old rodents present with lipofuscin granules, an insoluble, autofluorescent glycolipoprotein and by-product of lipid peroxidation, a reduction in cellular process complexity, altered granularity, increased mRNA levels of pro-inflammatory (TNF α, IL-1β, IL-6) and reduced levels of anti-inflammatory cytokines (IL-10, TGFβ1) compared to 2 month-old rodents (Ye and Johnson, 2001; Maher et al., 2005; Nolan et al., 2005).

The extent of microglia gene expression profiles and brain specific transcription patterns have also been examined in a systematic and comprehensive manner using the well-powered microarray technology. These studies have been able to monitor functional classes of genes showing altered expression in cognitively normal aging across the adult lifespan (age 20–99 years), and have reported that “*immune activation is a highly prominent feature of cognitively normal brain aging which additionally primes the brain for neurodegenerative cascades and cognitive decline*” (Lukiw, 2004; Cunningham et al., 2005; Perry et al., 2007; Berchtold et al., 2008; Holmes et al., 2009; Burger, 2010; Cribbs et al., 2012).

Cribbs et al. (2012) recently reported the response profiles of these genes implicated with brain aging, and these included a continuum of widespread upregulation in pro-inflammatory genes such as complement components, Toll like receptor signaling (TLRs), inflammasomes, scavenger and immunoglobulin (Fc) receptors and human leukocyte antigens I and II, reflecting activation of microglia and perivascular macrophages. Others have also reported upregulation in DAXX, a Fas binding protein, apoptosis mediator, cytosolic phospholipase A2, Nuclear factor κB (NF-κB), apoptosis signaling cell surface antigen TNFR family death receptor, Fas, IL-1β, IL-1α and cyclo-oxygenase-2 (COX-2) signaling in the hippocampus (see Colangelo et al., 2002; Lukiw, 2004). These changes were also coupled with a downregulation of select factors, such as: TOLLIP, fractalkine, IL-4, and CD200, C3CX3L1 which are known to be inhibitory to microglial/macrophage activation (Lukiw, 2004; Cribbs et al., 2012). Notably in all these gene expression profiling studies, essentially all pathways of the innate immune system were upregulated in aging, and this was associated with brain regions vulnerable to accumulating pathology in brain aging, namely the hippocampus, entorhinal cortex and superior frontal gyrus, all critical for higher cognitive function.

Such glial specific cell changes, however, do not occur in isolation, but likely in the context of increased cellular stress responses or deficiencies in cellular “coping mechanisms” that are associated with aging (Amenta et al., 1998; von Bernhardi et al., 2010). Collectively, glial cell activation in parallel with such physiological changes could be considered as the driving factors, although not exclusively (see Conde and Streit, 2006), contributing towards the cognitive decline seen in old age (Schipper, 1996; Simpson et al., 2010; von Bernhardi et al., 2010; Rodríguez and Verkhratsky, 2011), and therefore could be a potential target for preventive treatment, or “prophylactic” therapy.

### Microglia Senescence in Aging

Reports of extensively distributed senescent age-related dystrophic microglial cells have been shown throughout the brain in human subjects (Streit et al., 2004; Reichenbach et al., 2010). The morphological feature of dystrophic microglia as described by Striet et al., include: deramification or decreased arborisation of their processes, loss of finely branched cytoplasmic process, cytoplasmic beading/spheroid formation, shortened and twisted cytoplasmic processes and instances of partial or complete cytoplasmic fragmentation (Streit et al., 2004; Reichenbach et al., 2010).

Although the mechanisms involved in this characteristic age-related morphological transformations are unknown, it is believed that oxidative products that accumulate in microglia during aging (Hayashi et al., 2008; Lopes et al., 2008) make them vulnerable to oxidative damage and cell death. Microglial senescence might also result from a process involving telomere shortening, similar to immunosenescence of macrophages. Telomeres, which are the physical end of eukaryotic chromosomes continually shorten with age and divide due to the instability of DNA polymerase to completely replicate DNA molecules. Therefore when cells exhaust their replicated potential they enter replicative senescence, resulting in substantial damage to cell function and gene expression. Telomere shortening in rat microglia has been demonstrated over time *in vitro/in vivo* (Flanary and Streit, 2003). As microglial cells have a considerable capacity for cell division in the CNS (Clarke et al., 2008), a reduction in telomere length shortening could potentially damage their ability to combat pathological occurrence in the brain (Flanary et al., 2007). Senescence could cause microglia to function abnormally with an aberrant proliferative capacity, impaired clearance mechanism and reduced production of anti-inflammatory products. An increase in these distinct populations of dystrophic senescent microglial cells with age could therefore cause microglial cells to inevitably lose their vital homeostatic functional role in the CNS. This failure to respond adequately to external stimuli could thus eventually contribute towards neurodegeneration seen with aging (Streit, 2006; Streit et al., 2009).

### Glial Activation, Oxidative Stress and AGEs

Activated glial cells in the aged brain have been shown to increase their generation of by-products of oxidative metabolism including ROS—hydrogen peroxide, hydroxide ions, NO and peroxynitrite (Lynch, 1998b, 2010; Streit et al., 2004). These cytotoxic molecules can diffuse to other parts of the cell, damaging proteins, nucleic acids and lipids, causing oxidative stress, dysfunction of mitochondria and their membrane integrity, and subsequent activation of apoptotic mechanisms. One important by-product of glial oxidative metabolism is the production of *advanced glycation end-products* (AGEs), which are proteins that form non-enzymatically in a reaction between reducing sugars, amino groups of proteins and other compounds. AGEs are known to increase in aging in humans and rodents (Kimura et al., 1996, 1998) and engagement with the AGE receptor (RAGE) expressed prominently on microglial cells can activate the common NF-kappaB signaling pathways that is involved in stimulating oxidative stress, apoptosis and pro-inflammatory responses (Kimura et al., 1996, 1998).

In addition to the increase in by-products of oxidative metabolism, glial cells particularly astrocytes produce antioxidant glutathione and supply their precursors to neurons. This pivotal oxidative stress defense enzyme is known to greatly decrease with aging in the brain and plasma, resulting in the susceptibility to oxidative stress (Butterfield and Sultana, 2007). Such age-related peculiarities in anti-oxidant protection can be partly attributed to the switch in the metabolic support of astroglia to inflammatory cells capable of inducing neuroinflammation through production of pro-inflammatory and cytotoxic factors (Fuller et al., 2009).

### Microglia Priming in Aging

In the aged hippocampus of rodents, microglia activated by single acute exposure to small concentrations of systemic LPS (Henry et al., 2009; Frank et al., 2010) have been shown to exhibit an increase in mRNA and protein expression of pro-inflammatory genes (IL-1β and IL-6, CD11b, MHCII, Toll-like receptor, indoleamine 2,3 dioxygenase) and reduced anti-inflammatory cytokine levels compared to age-matched controls.

Although humans and animals are rarely exposed to a single acute systemic inflammatory event, such as, sepsis, they can however, encounter low concentrations of inflammatory toxins over a prolonged period. Perry and Colleagues recently demonstrated a similar effect on glial cell responses in the brains of rodents exposed to long-term systemic inflammation (Püntener et al., 2012). In a set of elaborate experiments by Püntener et al. (2012) it was demonstrated that injections with bacterial infectious agent *Salmonella typhimurium* was able to cause acute, transient behavioral changes and a robust peripheral immune response, peaking at day 7 after treatment. This resulted in a delayed and prolonged cytokine (IL-1β, IL-12, TNF-α) synthesis, followed by an activated microglia and cerebrovascular phenotype persisting until 4 weeks post infection in the brain of aged animals compared to age-matched controls. In addition, subsequent focal inflammatory challenges with LPS in these animals were able to prime brain innate immune responses, causing hyper-induction of pro-inflammatory molecules. These studies provide convincing evidence that suggests the inflammatory status of aged microglia is (hypo)tolerant and (hyper)primed to become inevitably over responsive to low-grade stimuli, such as, acute stress-related systemic inflammation experienced throughout life.

## Age-related Neuroinflammation and Impact on Hippocampal Neurogenesis

In the adult mammalian brain, located within the subgranular zone (SGZ) of the dentate gyrus are neuronal progenitor cells, which generate thousands of new neurons on a daily basis (Altman and Das, 1965; Cameron et al., 1993; Kuhn et al., 1996). These newly generated post-mitotic neurons, extend their axons rapidly and migrate to the granule cell layer after cell division, where they mature to develop a neuronal phenotype and morphology. Some emerging evidence suggests that neurogenesis plays a pivotal role in hippocampal function, particularly in memory consolidation and spatial learning (Shors et al., 2001). Impairment in hippocampal neurogenesis may therefore be linked to cognitive decline. A number of studies have shown that neurogenesis in the hippocampus dramatically declines with age (Seki and Arai, 1995; Kuhn et al., 1996; Rao et al., 2005; Olariu et al., 2007). This reduced potential of the aged brain to generate a constant rate of neuronal cell turnover has been attributed to age-related neuroinflammation. This role was elegantly demonstrated in an *in vivo* study showing that LPS induced inflammation gives rise to microglia activation in the close proximity of newly formed neurons in the SGZ, and strongly impaired basal and insult triggered neurogenesis reflected by a reduction in BrdU/NeuN+ immature neurons in rats (Ekdahl et al., 2003). Moreover the impaired neurogenesis associated with inflammation was restored by systemic administration of minocycline, which inhibits microglia activation, suggesting that impairment in neurogenesis depends on the degree of microglia activation. The mechanism(s) by which age-related localized neuroinflammation in the hippocampus compromises the survival of newly born hippocamapal neurons is currently unknown. However, the role of releasable cytokines, such as IL-1β, IL-6, NO, TNFα from localized microglia and astrocytes (Ekdahl et al., 2003; Hanisch and Kettenmann, 2007) and diminished production of growth factors such as brain-derived growth factor (BDNF) and fibroblast-derived growth factors (FGF) primarily produced by hippocampal astrocytes in the dentate gyrus has been proposed to impact on local and systemic external factors in the neurogenic zone (niche). Consequently, impairing the activation of quiescent stem cells, the mitotic potential of proliferating precursors, and the survival of neuronal fate-committed precursors.

## Age-related Neuroinflammation and Impact on Vascular Dysfunction and Associated Comorbidities

A dynamic network of microvessels function to meet the brain metabolic demands. This vital neurovascular unit comprises of endothelia cells, astrocytic end-feet processes, pericytes, smooth muscle cells, neurons and neighboring perivascular microglia processes. Impairments in cerebrovascular function therefore carries with it detrimental consequences. It is increasingly evident that there are subtle structural changes to cerebral microvascular integrity in the brains of non-demented elderly individuals without overt concurrent neuropathology. Some of these significant age-related impairments in the vascular network of small vessels, such as, arterioles and capillaries of the basal forebrain regions and the hippocampus are characterized by; (i) reduced vessel density; (ii) venous collagenosis; (iii) tortuosity lesions; (iv) thickened arteriolar and capillary walls/arteriosclerosis; and (v) deposition of lipofusin, dystrophic, calcified glial elements or corpus amylacea and in some cases cerebral amyloid angiopathy (CAA) around blood vessels (Braak, 1979; Scheibel, 1984; Mrak et al., 1997; Esiri et al., 1999; Breteler, 2000; Esiri, 2000; Weller et al., 2002; Love et al., 2003; Vasilevko et al., 2010). These data together indicate that vascular deterioration in the aged brain appears to have strong implications for neurodegeneration, and affect longitudinal cognitive trajectories adversely, perhaps leading to cognitive decline in aging (Richardson et al., 2012; Vemuri et al., 2015). The consequences of such deficits in vascular integrity could manifest itself by causing the loss of vascular auto-regulation associated with rigidity of arterioles and insufficient perfusion of branching vessels in the territory of susceptible areas such as the temporal cortex and hippocampus (Popa-Wagner et al., 2014). Functional neuroimaging studies that detect cerebral blood flow and integrity have provided conspicuous evidence that corroborates a slow but progressive systematic breakdown of cerebral vascular perfusion with aging (Martin et al., 1991).

Such deficits in vascular perfusion among the elderly have been linked with other age-related comorbidities such as vascular dementia associated with CAA and late-life vascular depression (Brevik et al., 2013; Muresanu et al., 2014). Intriguingly the latter has been shown to greatly increase the risk of cognitive decline in aged individuals (Lenze et al., 2005), including other age-related diseases affecting the cardio*vascular*, cerebro*vascular*, neuro-endocrine and immune systems (Popa-Wagner et al., 2014). Depression has long been associated with increased markers of inflammation (such as: IL-6, IL-8, IFN-γ, TNF-α, CRP) in the periphery (Popa-Wagner et al., 2014). In such circumstances it is possible that on the background of systemic inflammation with aging, where there is already a chronic low-grade pro-inflammatory state in the brain, pro-inflammatory cytokines in the periphery may additionally gain access to the CNS and trigger microglial activation and subsequent neuroinflammation (Dilger and Johnson, 2008; Ransohoff and Cardona, 2010; Popa-Wagner et al., 2014). This in turn could consequently result in a self-perpetuating event involving further activation of inflammatory responses in the brain and precipitation of depressive-like symptoms and cognitive decline.

Additionally, age-related comorbid vascular changes are commonly reflected in regional and laminar patterns that parallel patterns of perivascular or localized parenchymal inflammation (Buée et al., 1994; Richardson et al., 2012). Consistent with this histopathological outcome, mouse models subjected to chronic cerebral hypoperfusion also intriguingly display marked increase in vascular/perivascular infiltration of inflammatory cells (Reimer et al., 2011; Okamoto et al., 2012). As a result, underlying age-related neuroinflammatory processes (in the brain and also periphery) have been considered to be a potent contributor (and/or a predisposing factor) for age-related microvascular damage. With the vascular brain endothelium and associated cells thought be the point of convergence for mechanisms of inflammatory-associated damage. This may occur through the accumulative effect of low grade inflammatory factors, such as: nitric oxide (NO), thrombin, tumor necrosis factor-α (TNFα), transforming growth factor-b (TGF-β), interleukin (IL) IL-1β, IL-6, IL-8 (NFκ-B signaling), VCAM, ICAM and matrix metalloproteinases (MMPs) released from activated glial and perivascular cells, and impacting on microvascular function. We postulate that the outcome of these responses in the brain could lead to: (i) alterations in cerebral blood flow/perfusion through inflammatory vasodilators; (ii) activation of death signaling pathways in endothelial cells, smooth muscle cells, and pericytes; (iii) providing signals for perivascular cell invasion into the brain; (iv) disruption of BBB integrity; and (v) alterations in perivascular drainage/clearance (or CSF turnover) through abluminal transport mechanisms. These mechanisms in aging have not been greatly explored and should be considered in future preclinical studies.

## Role(s) for Neuroglial Cells in Synaptic Function and Age-Related Spatial Learning Deficits

Another aspect of neuron-glia interaction often overlooked and which is of vital importance to normal physiology is the support of synaptic function, in which astroglia and microglia are of particular importance.

The elaborate dynamic processes of resting microglial cells have been shown by *in vivo* live imaging studies to contact synapses vigilantly monitoring and responding to biochemical perturbations (Wake et al., 2009). For example resting microglia processes under physiological conditions reportedly made brief but frequent direct contacts at synapses lasting 5 min, however in conditions whereby neuronal activity was upregulated the frequency of contacts was reduced, with direct contacts lasting for 1 h. In pathological cases the spatio-temporal interaction between microglia and synapses was even more markedly prolonged and occasionally followed by the disappearance of synaptic structures i.e., boutons and spines (Trapp et al., 2007; Wake et al., 2009; Tremblay et al., 2010). This unique role of microglia clearance has been suggested to involve components of the MHC class I molecule and proteins from the complement cascade, for example C1q and C3-CR3/CD11b-CD18/Mac-1 complement-phagocytic receptor complex localized to synaptically enriched regions, which have been documented to selectively tag synapses in the developing visual system at a time when refinement and synaptic remodeling and plasticity of neuronal circuits occur. A number of studies have reported that during aging there is a loss of synaptic contacts in the CNS especially in the hippocampus (Geinisman et al., 1986a,b; Nicholson et al., 2004) accompanied by an underlying microglial activation displaying increase phagolysosomal vesicles and an increase in microglia-synaptic interactions (Ojo et al., 2012, 2013). Primed/activated microglia cells may therefore have a major role in the elimination and disruption of these synaptic connections with aging (Ojo et al., 2012, 2013) and studies to examine the cellular and molecular cascades involved would be important in order to understand the contribution of microglial cells to the plasticity of neuronal circuits in the aging brain.

Several neurochemical parameters in the hippocampus implicated in synaptic plasticity, including learning and memory processes, are sensitive to immuno-active molecules produced primarily by microglia and to a lesser degree by astrocytes. Cognitive function is disrupted by neuroinflammation following IL-1β, IL-18, and LPS treatment in rodent models. For example, treatment with pro-inflammatory cytokine IL-1β, which increases notably with aging or products that increase its levels indirectly, inversely correlates with the ability of rats to sustain long-term potentiation LTP, a classical paradigm of spatial memory (see Lynch, 1998a). IL-1β disrupts LTP via activation of IL-1 type I receptor signaling (IL-1R1) (expressed at a high density in the hippocampus) and through the phosphorylation of stress-activated protein kinase, c-jun N-terminal kinase (JNK) death associated cellular pathway (Barry et al., 2005; Moore et al., 2007). IL-1β can also stimulate lipid peroxidation through production of free radicals, consequently decreasing membrane polyunsaturated fatty acids, such as arachidonic acid, a well known putative retrograde messenger involved in synaptic plasticity and the expression of LTP (Bliss and Collingridge, 1993). This peroxidative process subsequently alters membrane fluidity and induces biophysical changes on membrane associated functions, such as: impaired ion channels-pumps and enzymatic activity of lipases/kinases, reduced receptor density and associated functions (Lynch, 1998b) and impairment in other protein-protein interactions such as neurotransmitter release (Murray et al., 1997). Notably some of these molecular consequences and functional deficits can be rescued by anti-inflammatory agents such as IL-4, CD200, IL-10, minocycline and eicosapentaenoic acid (EPA), which are inhibitors of glial activation (Lynch, 1998b; Kelly et al., 2003; Lyons et al., 2007a,b; Minogue et al., 2007; Clarke et al., 2008). It is therefore likely that the imbalance between pro-inflammatory and anti-inflammatory cytokines in the primed aged brain, perhaps also attributed to by an imbalance in the periphery, contributes significantly to age-related deficits in cognitive function.

To support these changes observed in rodent models, deficits in cognitive functions associated with inflammation have been ostensibly reported in human patients receiving cytokine treatment for cancer and hepatitis (Griffin et al., 2006; Ownby, 2010). Also, systemic influenza infections induced by active systemic inflammatory substances that are capable of opening the BBB specialization have been linked to neurobehavioral dysfunction including cognitive deficits and also lethargy, increased sleep/narcolepsy and social withdrawal (Ownby, 2010). However, whether the neuroinflammatory effects of influenza are a direct (i.e., primary) or indirect (i.e., secondary) causative factor on cognitive dysfunction is yet to be determined at the preclinical level.

Astrocytes are also known to be in close proximity to synaptic structures. In the hippocampus it is thought that over 60% of all axon-dendritic synapses are surrounded by finely branching astroglial membrane processes, with peculiar specificity for large perforated synapses (~80%), which are presumably the most functionally active, and <50% of smaller (macular) synapses (Bushong et al., 2002; Ogata and Kosaka, 2002; Halassa et al., 2007). A single protoplasmic astrocyte is estimated to contact several hundred dendrites from multiple neurons and can envelope 100,000 or more synapses (Sofroniew and Vinters, 2010). This intimate astroglial connection with synapses is thus very important for support towards a host of synaptic functions which include: (i) control of synaptogenesis, dendritic spine cytoskeletal stabilization and remodeling (Murai et al., 2003); (ii) ionic homeostasis; (iii) neuronal excitability; (iv) energy support for neurons (Brown et al., 2004; Kofuji and Newman, 2004; Pellerin et al., 2007); (v) synthesis, uptake and recycling of glutamate (Hertz and Zielke, 2004); and (vi) responding to synaptic activity through bi-directional release of astrocytic-secreted factors, synaptically active molecules or gliotransmitters including glutamate, purines (ATP and adenosine), GABA, lactate and D-serine and cell-cell interaction with contact-mediated factors (Zhuo et al., 1993; Bezzi et al., 1998; Nishiyama et al., 2002; Zhang et al., 2003). Because synaptic-astroglial interactions are essential for healthy brain activity (Reichenbach et al., 2010), modifications in this intimate structural relationship may have a detrimental effect on the functional relationship between astroglial cells and synapses in the aging brain; most notably in glutamatergic circuits, which is particularly important in hippocampal dependent learning and memory processes.

In the hippocampus, astrocytes express glutamate receptors (mGluR, NMDA and AMPA receptors) and can respond to changes in neuronal glutamate levels (Sofroniew and Vinters, 2010). It has been speculated that astrocytes are able to synchronize with neuronal activity by sensing local changes in glutamate concentrations at glutamatergic synapses through their receptors, resulting in the vesicular release of gliotransmitters such as glutamate, ATP and D-serine (a co-agonist of NMDA receptors) via exocytosis involving calcium dependent mechanisms. These gliotransmitters bind to their respective receptors to regulate influx of ions into postsynaptic neurons and subsequent neuronal depolarization that initiates the induction of LTP. This involves a cascade of molecular mechanisms that stimulate the transient and persistent activation of kinase enzymes, such as: calcium calmodullin dependent protein kinase II (CaMKII), protein kinase A and C (PKA, PKC) and mitogen activated protein kinase (MAPK) that converges to initiate transcription factors (CORT1, CREB), and the expression of synaptic plasticity during learning and memory formation (Lynch, 1998a). Electron microscopy studies have reported increased occurrence of perisynaptic astroglia in tissue samples exposed to induction of LTP (Lushnikova et al., 2009), whereas a memory consolidation task appeared to decrease synaptic astroglial coverage (Ostroff et al., 2014). We recently reported in a detailed morphometric study that these astrocyte-synaptic interactions are significantly reduced in the aged rat hippocampus (Ojo et al., 2012). We speculate that during aging the astrocytic change from resting-quiescent into a mild-to-moderate activated or hypertrophic state could cause astrocytes to relinquish some of their neurosupportive roles at the synapse. Possibly dampening down their ability to sense incoming neuronal activity and reducing their production and/or release of important gliotransmitters, thus resulting in an impairment in the regulation of synaptic activity, information processing at glutamatergic neural circuits and hence age-related deficits in learning and memory processes.

In addition to directly releasing glutamate and related co-agonists at glutamatergic synapses, astrocytes are also paramount in regulating concentrations of glutamate levels at the synapse, during neuronal activity. Uptake of synaptic glutamate is a major mechanism that prevents accumulation in the synaptic space and thus protects against excessive activation and excitotoxic cell death (Fuller et al., 2009). Astrocytes express the glutamate transporter EAAT2 or GLT1, which are a main regulator of extracellular glutamate levels, and also contain enzyme glutamine synthetase, which amidates glutamate to glutamine in an ATP-dependent manner for recycling by neurons. Notably EAAT2 levels and gliosis were shown to inversely correlate in the aging brain (Simpson et al., 2010), indicating that the hypertrophic astrocytes in the aging brain may lose their expression of EAAT2, with a resultant deficit in regulating glutamate levels during synaptic activity.

Another important and often ignored gliotransmitter released by astrocytes is lactate. During the process of memory formation, which involves a cascade of cellular and molecular processes, to ensure adequate functionality of neuronal activity, astrocytes provide neurons with lactate, a useable and preferred energy substrate. Lactate is produced by a process of glycogenolysis. Astroglia end feet processes take up glucose transported across the BBB via glucose transporter (GLUT) 1. Glucose is either stored as glycogen or metabolized by glycolysis involving action of lactate dehydrogenase (LDH) isoenzymes LDH5, resulting in the production of lactate, which can be released into the synapse through the mono-carboxylate transporters 1 and 4 (MCT1, MCT4; Ota et al., 2013). During synaptic activity neurons take lactate via an MCT2 transporter and convert this into pyruvate for oxidative metabolism (Ota et al., 2013). This mechanism is supported by studies in rats showing that inhibition of glycogenolysis and transporters MCT1, MCT4 or MCT2 can induce memory impairment in inhibitory avoidance and hippocampal dependent spatial memory tasks (Newman et al., 2011; Suzuki et al., 2011). Because synaptic activity is coupled to energy utilization through this astroglial-neuronal interactive mechanism, involving metabolism of astrocytes, we hypothesize that during aging this astrocyte-neuronal lactate shuttle may also become significantly impaired due to a reduced metabolic support of astrocytes in their hypertrophic state.

In addition to regulating synaptic activity through gliotransmitters, astrocytes also have the potential to exert powerful and long-term influences on synaptic function through the release of growth factors and related molecules such as nerve growth factor (NGF), basic fibroblast growth factor (FGF), transforming growth factor-Beta (TGF-β), platelet-derived growth factor (PDGF), brain-derived neurotrophic factor (BDNF), cilliary neurotrophic factor (CNF), survival-activity dependent neurotrophic factor (ADNF) and cytokine TNF-α (Schwartz and Nishiyama, 1994; Rudge et al., 1995; Dreyfus et al., 1999; Messersmith et al., 2000; Albrecht et al., 2002).

LTP and memory consolidation have been shown to involve the contributory roles of growth factors and cytokines, which are highly expressed along with their receptors in the hippocampus (Jankowsky and Patterson, 1999). These proteins can alter neuronal morphology, gene expression and proliferation. The best-studied growth factor is brain derived neurotrophic factor (BDNF), highly concentrated in the hippocampus (Wetmore et al., 1990; Murer et al., 2001). BDNF is largely produced by astrocytes and is important in synaptic plasticity (and also neurogenesis; see section Age-related neuroinflammation and impact on hippocampal neurogenesis). It has been shown to be an extremely potent modulator of theta burst induced LTP, involving communication with the extracellular signal regulated kinase (ERK) and p38 MAPK pathways and phosphorylation of cAMP response element binding protein (CREB) transcription factor, implicated in late phase LTP, gene transcription of activity-regulated cytoskeleton-associated protein, Arc, protein synthesis and spine-dendritic plasticity (Ying et al., 2002; Rex et al., 2007). BDNF has been shown to decrease in late adulthood in correlation with a decline in hippocampal volume (Lommatzsch et al., 2005; Ziegenhorn et al., 2007; Erickson et al., 2010). FGF, another growth factor largely produced by hippocampal astrocytes located in the dentate gyrus has also been largely studied, and its receptor FGFR1 has been demonstrated to be required for LTP and memory consolidation, promoting neurite outgrowth and enhancing synaptic connectivity (Zhao et al., 2007). FGF-2 has been shown to also significantly decrease with aging (Shetty et al., 2005).

Astroglial derived growth factor ADNF and cytokine TNF-alpha have been demonstrated to influence homeostatic synaptic scaling by regulating glutamate release and insertion of AMPA and NMDA receptors in the membrane of neighboring neuronal synapses respectively (Blondel et al., 2000; Ota et al., 2013). These mechanisms are essential for the maturation of synapses and the maintenance of the post-synaptic density. Astroglial also produce and release cholesterol, which is indispensable for synaptic formation, providing building materials for new membranes, also acting as precursors for conversion into neuroactive steroid hormones that function as synaptogenic signals. Several other astroglial factors including agrin and ephrin signaling have also been implicated in regulating synaptic function, stimulating and inhibiting synaptogenesis and dendritic outgrowth respectively through actin rearrangement (Ota et al., 2013). We hypothesize that the disruption of these pleiotropic neurosupportive roles of astrocytes mediated by the exhaustive lists of secreted membrane bound or releasable astroglial factors with aging may carry serious deleterious consequences on synaptic and other physiological functions.

## Neuron-Microglia Interactions and Glial Inhibitory Factors in Aging

Neurons are now accepted as vital inflammatory/glial cell modulators in the brain. They have been shown in different experimental/clinical settings to control astrocyte and microglial activity. Several neuronal signaling molecules including TREM2 and CD200, which have both received a wide variety of attention in the past few years have been proposed to play a prominent surveillance role in influencing the inflammatory milieu of the CNS (Biber et al., 2007). TREM2 is an anti-inflammatory ligand and danger associated molecular pattern protein (DAMP) that controls CNS immune homeostasis by interacting with its innate immune receptor TREM2R on microglia cells. Specifically, TREM is released from apoptotic neurons and controls microglial phagocytosis and clearance. Mutations of TREM2 exhibit the pathological hallmarks of the neurodegenerative disease Nasu Hakola or PLOSL (Takahashi et al., 2005; Neumann and Takahashi, 2007). No studies to date have established a link between aging and TREM2 dependent microglial function. It is possible that the underlying accumulation of protein aggregates present in aged brains (Davis et al., 1999) are by-products of an ineffective role of TREM2 dependent signaling in controlling microglia clearance of potentially toxic cellular debris. Other cell surface factors such as low-density lipoprotein receptor (LDL-R), LDL receptor related protein-1 (LRP1), alpha-macroglobulin, scavenger receptor—A1 (SR-A1) and Formyl peptide receptor like receptor (FPRL) implicated in microglia clearance mechanisms may also be implicated.

Neuronal CD200 is a glycoprotein that interacts via cell-cell contact with its cognate receptor (CD200R) localized mainly on cells of the myeloid lineage including microglial cells (Barclay et al., 2002). Activation of CD200R has been shown to spatially modulate the extent of glial activation by induction of quiescence (Lyons et al., 2007a). A number of studies have provided substantial evidence that the absence of CD200-CD200R interaction results in the damage and loss of neurons, and hyper-responsiveness of glial cells leading to a neurodegenerative pathology and functional impairment (Popovich and Longbrake, 2008). CD200 knock out mice have been shown to exhibit sporadic activation of glial cells (Broderick et al., 2002) and antibody-mediated blocking of CD200 in *in vivo* and *in vitro* models have demonstrated evidence of neurodegeneration (Meuth et al., 2008). Blocking of CD200R *in vivo* also augmented pathological/clinical signs of experimental autoimmune encephalomyelitis (EAE) in rodents (Hoek et al., 2000; Broderick et al., 2002). Consistent with these findings, a deficit in LTP induced by LPS stimulation was rescued by intra-cerebroventricular injections of CD200Fc immunoglobulins (Lynch, 2010; Cox et al., 2012). These studies highlight the pivotal role of CD200 in protecting against inflammatory reactions in the normal healthy brain, where its levels are reportedly reduced (Ojo et al., 2012; Cox et al., 2012). Low levels of CD200 (including other glial inhibitory factors, such as, CX3CL1:CX3CR1) could therefore contribute to the primed neuroinflammatory state of glial cells frequently reported in aged brains.

## Conclusion

**Figure 2:**
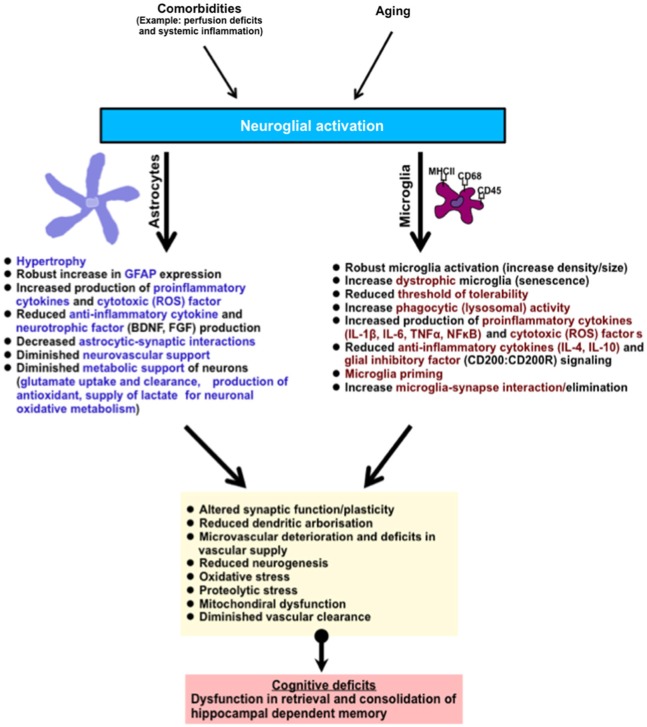
**Summary of age-related neuroglial response(s) in the brain (hippocampus)**. The hippocampus is involved in the retrieval and consolidation of memory, and with advanced aging hippocampal function progressively deteriorates. These changes correlate with underlying hallmark neuroglial cell response, typified by the activation of microglial and astroglial cells, robust morphological changes, and an increase in phenotypic expression of antigen activation markers (MHCII, CD68, CD45, GFAP). In addition, activated neuroglial cells in aging also display increased production of pro-inflammatory cytokines and their signaling profiles, phagocytic/lysosomal activity, toxic oxidative-lipid products and a reduction in the production of anti-inflammatory cytokines and neurotrophic factors. In microglial cells, these changes are thought to contribute to their priming (and possible senescence), reducing their threshold of tolerability and therefore shifting the balance to increased age-related neuroinflammation and hence neurotoxicity. Astroglial cells, which are essential in metabolic support of neurons and vascular cells, progressively relinquish these supportive roles leading to deterioration in brain function. Together this culminates in subtle impairments in dendritic integrity, synaptic function, neurogenesis, increased oxidative metabolism, diminished protein clearance, and microvascular deterioration. Abbreviation: (ROS) reactive oxygen species such as nitric oxide, superoxide.

The hippocampus is one of the brain regions that are vulnerable to age-related deterioration. It is constantly engaged in the spatial processing of episodic and declarative memory of information throughout life. An underlying neuroinflammatory response in the hippocampus, typified by astrocyte and microglial activation and an increased pro-inflammatory cytokine production is one of the major hallmarks of aging. This may occur without concurrent incipient pathology. Neuroinflammatory cells in this state could function abnormally losing their quiescent mechanisms, becoming over-responsive to stress or/stimuli, producing neurotoxic and proinflammatory molecules, and surrendering pivotal metabolic and neurosupportive roles, ultimately altering important physiological functions (see summary Figure 2). A major challenge for future consideration in this field is to determine what factors are involved in maintaining neuroinflammatory cells in a quiescent non-inflammatory state in the brain, and whether pharmacological manipulations might be important in preserving hippocampal and hence cognitive function in aged individuals.

## Conflict of Interest Statement

The authors declare that the research was conducted in the absence of any commercial or financial relationships that could be construed as a potential conflict of interest.
